# Canada’s Medical Assistance in Dying System can Enable Healthcare Serial Killing

**DOI:** 10.1007/s10730-024-09528-3

**Published:** 2024-08-02

**Authors:** Christopher Lyon

**Affiliations:** 1 Department of Environment and Geography, University of York, York, YO10 5DD UK; 2 Leverhulme Centre for Anthropocene Biodiversity, University of York, York, YO10 5DD UK

**Keywords:** Assisted suicide, Medical assistance in dying, Euthanasia, Serial murder, Healthcare, Medicine, Criminal law, Risk management

## Abstract

The Canadian approach to assisted dying, Medical Assistance in Dying (MAiD), as of early 2024, is assessed for its ability to protect patients from criminal healthcare serial killing (HSK) to evaluate the strength of its safeguards. MAiD occurs through euthanasia or self-administered assisted suicide (EAS) and is legal or considered in many countries and jurisdictions. Clinicians involved in HSK typically target patients with the same clinical features as MAiD-eligible patients. They may draw on similar rationales, e.g., to end perceived patient suffering and provide pleasure for the clinician. HSK can remain undetected or unconfirmed for considerable periods owing to a lack of staff background checks, poor surveillance and oversight, and a failure by authorities to act on concerns from colleagues, patients, or witnesses. The Canadian MAiD system, effectively euthanasia-based, has similar features with added opportunities for killing afforded by clinicians’ exemption from criminal culpability for homicide and assisted suicide offences amid broad patient eligibility criteria. An assessment of the Canadian model offers insights for enhancing safeguards and detecting abuses in there and other jurisdictions with or considering legal EAS. Short of an unlikely recriminalization of EAS, better clinical safeguarding measures, standards, vetting and training of those involved in MAiD, and a radical restructuring of its oversight and delivery can help mitigate the possibility of abuses in a system mandated to accommodate homicidal clinicians.

**Supplementary Information:**

The online version contains supplementary material available at 10.1007/s10730-024-09528-3.

## Introduction

While source definitions vary, serial killing (SK) is generally defined as the homicide of two or more people on discrete occasions (rather than collectively or mass) for reasons that may be related to the killer’s personal views, material, or psychological benefit (Adjorlolo & Chan, 2014; Miller, 2014a). The United States Federal Bureau of Investigation defines serial murder simply as “the unlawful killing of two or more victims by the same offender(s), in separate events,” regardless of motive (Morton & Hilts, 2005, p. 9).

Recorded since the nineteenth century (Kinnell, 2000), the SK subtype of healthcare serial killing (HSK) is defined as the intentional, individual and sequential killing of “helpless or dependent persons under their care” by medical workers, often physicians and nurses (Miller, 2014a, p. 6). Rationales or motives for HSK include perceiving vulnerable, elderly, sick, or disabled people as burdens or in need of death to relieve suffering and to provide dignity and may still overlap with general SK motives of sadism and material or financial gain (Miller, 2014a; Tang, 2020; Yorker et al., 2006).

Criminal HSK appears globally. Offenders may kill fewer than ten to hundreds of people. They are often challenging to detect and stop due to job-related access to means of killing, responsibility for record-keeping, trusted role, professional insularity and protectionism, poor oversight, and victims whose deaths are less likely to attract suspicion due to age, illness, or existing likelihood of dying (Foong-Reichert et al., 2021; Lubaszka et al., 2014; Tilley et al., 2019; Yorker et al., 2006). Ambiguous or divergent legal and medical concepts and practices can make prosecuting HSK challenging (Crofts, 2022).

Medical Assistance in Dying (MAiD), the expression frequently used to describe legal euthanasia or assisted suicide (EAS) in Canada^1^ and elsewhere, is currently available in several countries and under consideration in others (British Medical Association, 2021). Beginning in 2016 with “no model…without standards, without guidance, without training” (The Fifth Estate, 2023), Canada’s MAiD system is criticized as the most permissive or least safeguarded in the world (Briscoe & Widera, 2024; Scott & Scott, 2023), raising the question of whether it could protect patients who fit the clinical profile of adult victims of HSK from a killer working as a MAiD provider. Indeed, like the systemic issues that enable criminal HSK, concerns are frequently flagged that the risks stemming from the Canadian MAID programme’s ambiguous criteria, noncompliance with law and regulation, applications in mental illness, impact on clinical staff, and inconsistent oversight (Chochinov et al., 2023; Christie & Li, 2023a; Coelho et al., 2023; Gaind et al., 2022; Gaind, 2023; Koch, 2022; Kono et al., 2023; Lane, 2023; Lyon, 2024; Pullman, 2023b). Accordingly, assessing the Canadian system through the HSK lens is helpful in illuminating gaps in the safeguards and opportunities to prevent abuses by such an offender. This task is critical, as forms of MAiD are available or under consideration in many countries, and Canadian courts seem likely to maintain some form of its constitutional permissibility (Lemmens, 2023).

While there is substantial legal, criminological, and bioethical literature on illegal euthanasia and assisted suicide (EAS) in non-MAiD jurisdictions, this paper contributes to the currently sparse scholarship exploring HSK *within* legalized MAiD regimes (Crofts, 2022). As such, it tests and challenges assertions that they are “strongly slanted against sinister manipulation” (Nielsen, 2021, p. 95), responds to calls for more explicit safeguards as MAiD legalization and expansion increase globally (Richardson, 2023), and helps us answer the outstanding question of who should (not) provide MAiD (Schipper, 2020). Consequently, it will be of value to legal and law enforcement authorities, healthcare system risk managers, policymakers, insurers, researchers, and others in all MAiD jurisdictions.

The paper begins with a brief account of MAID’s history and situation as of early 2024, followed by a discussion of terminology. It then explores the structure and parallels between MAiD and serial murder opportunities in Canada and concludes with recommendations for better safeguards.

## MAiD in Canada

MAiD has been legal since 2016 through two criminal law amendments resulting from court cases (Criminal Code, 1985). Other sources discuss this background at length (Downie, 2022; Dumsday, 2023; Grant, 2023; Koch, 2022; Lemmens, 2018, 2023; Pullman, 2023b; Shariff et al., 2023), so only some pertinent issues are reviewed here.

The Criminal Code defines MAiD as a “non-culpable” form of homicide that is “not an offence,” whereas “culpable homicide is murder, manslaughter, and infanticide.” Similar to other countries (Downie et al., 2022),^2^ clinicians engaged in legal MAiD must assess candidates and terminate their lives within the legislated eligibility criteria. Track 1 MAiD, legal since 2016, is for people whose “natural deaths are reasonably foreseeable”. Since 2021, track 2 has been available for those with a “reasonably foreseeable natural death” after a 90-day “assessment period”.

Currently, to be assessed for legal MAiD, a person must be at least 18 years of age and capable of “making decisions with respect to their health” and independently assessed by two nurse practitioners or medical doctors to “have a serious and incurable illness, disease or disability,” to be in “an advanced state of irreversible decline in capability,” and that those issues produce “enduring physical or psychological suffering that is intolerable to them and that cannot be relieved under conditions that they consider acceptable” (Criminal Code, 1985, sec. 241). The applicant must also be informed of means to relieve their suffering. Still, such means do not have to be provided or attempted before death is available, a feature unique to Canadian MAiD.

The options for MAID are euthanasia (clinician-administered death by lethal injection) and assisted suicide (patient-administered death). While euthanasia is the only legal option in Québec (Health Canada, 2023), self-administration is often unoffered in other places (e.g., Saskatchewan Health Authority, 2023; The Ottawa Hospital, 2018). There is no waiting period for track 1 patients (a 10-day reflection period was discarded in 2021), and euthanasia may occur as soon as a person passes two assessments or anytime thereafter, pending the availability of a provider and lethal substances. Unlike other countries (e.g., 6 months per Oregon and New Zealand) (British Medical Association, 2021; Li, 2023), there is no time limit for a “reasonably foreseeable” death prognosis for track 1. For track 2 patients, a 90-day assessment period formally only requires passing the same two assessments and can be shortened for an imminent loss of capacity or foreseeable death. One of the track 2 assessors needs expertise (not necessarily a regulated specialism) in the patient’s cause of suffering or “consult” with a third practitioner who does (Criminal Code, 1985).

Track 1 eligibility is broad. It ranges from a clinically assessed terminal or worsening of a patient's diagnosed illness, disease, or disability that makes their death subjectively “reasonably foreseeable” (Criminal Code, 1985) to, at least as advised by the Canadian Association of MAiD Assessors and Providers (CAMAP), a remediable *intention* or *action* to attempt suicide through refusals of care, sustenance (i.e., voluntarily stopping eating or drinking, or VSED) or other unspecified measures that would cause or hasten death if left unremedied (CAMAP, 2022b; Lyon, 2024).^3^ Some published witness accounts and parliamentary committee testimony suggest the track 2 assessment period can be shortened this way (Parliament of Canada, 2023; Serota et al., 2023). Comments in CAMAP’s 2018 conference report and a journal also assert that a MAiD clinician is unlikely to be prosecuted for mentioning to an ineligible person forms of suicidal self-harm to hasten death or make them eligible for MAiD (CAMAP, 2018; Downie, 2018),^4^ implying that clinicians have an informal power to engineer eligibility (Lyon, 2024). At least one jurisdiction permits (or permitted) family members, not criminally exempted clinicians, to administer the lethal injections (Thomas et al., 2023). Québec’s MAiD commission annually identifies multiple cases of criminal and provincial law-breaking in MAiD deaths (Commission sur les soins de fin de vie, 2022; 2023), amid other cases in prisons, Ontario (Coelho et al., 2023) and British Columbia (Cook, 2023). However, there are no known instances of regulatory sanctions, prosecution, pending or attempted, of these transgressions, which include approvals and deaths of patients who lacked capacity or did not meet other eligibility criteria. A lawyer in an unsuccessful recent case challenging a MAiD approval pointed out that in their province “AHS [Alberta Health Services] operates a MAID system with no legislation, no appeal process and no means of review” (Grant, 2024).

The Canadian approach is sometimes called a “joint view” of assisted death, where the law tries to balance clinical judgment with patient autonomy and relative vulnerability (Braun et al., 2024; Davis & Mathison, 2020; Lazin & Chandler, 2023). However, researchers and current and former MAiD practitioners argue that the law is too subjective and lacks adequate oversight, leaving clinicians with significant practical authority to apply their values and ideas to MAiD cases (Christie & Li, 2023a; Gaind, 2023; Kotalik, 2023b; Lyon, 2024).

However, before the court rulings (*Carter v Canada (Attorney General)*, 2015; *Truchon and Gladu v Attorney General (Canada) and Attorney General (Quebec)*, 2019) that led to decriminalization of EAS, there were accounts of illegal deaths. These were often linked to the advocacy group Dying with Dignity Canada (DWDC—now both a charity and a federal lobbyist; Registry of Lobbyists, 2023; Schreiber, 2024), other clinicians, families, and friends (Crawford, 1997; Dienesch, 2003; Hoy, 1998; MacDonald, 1999; MacFarlane, 1998; Pike, 1997; van Wageningen, 1992; Zurowski, 1991). For instance, in 1998, past DWDC executive director Cynthia St. John and founder Marilynne Seguin stated that DWDC “counselled” and or “cared for” over 2,000 people (“all over the world”) since the organization’s 1980 origin (Hoy, 1998; MacFarlane, 1998). Seguin claimed to know of 145 related deaths in 1992 (van Wageningen, 1992) to have been present at 200 such deaths (Crawford, 1997), “even assisting in some” (MacFarlane, 1998), which she remarkably admitted to a Senate committee on EAS in 1994 (Seguin & Campbell, 1994a). In her 1994 Senate testimony, Seguin claimed DWDC receive a monthly rate of “500…inquiries about the end of life, either for themselves or for a member of their families. In the last few years, about 30 percent of those calls have been initiated by health care providers” (Seguin & Campbell, 1994a, pp. 22, 23).

What is noteworthy about this testimony is that DWDC seems to have entertained requests by third parties (Seguin & Campbell, 1994a). Seguin described assessing requests personally according to her self-devised eligibility criteria, and that about 3% of these inquiries resulted in people who “actually take their own lives or are assisted in taking their lives,” and described deaths, including a young medical student, “Joshua,” and his worries about putting his parents in legal jeopardy, as well as other instances (Seguin & Campbell, 1994a, pp. 22–25, 1994b). Counselling or assisting these deaths is prima facie criminal as EAS/MAiD was illegal, so these news reports and testimony point toward a substantial semi-covert homicide or suicide assistance program involving healthcare workers, family members, and others, facilitated by DWDC. Little is known about these deaths beyond such general admissions, including the identities and the circumstances of the instigators, victims, or clinicians. DWDC is now a well-funded and influential charity and federal lobbyist for MAiD (Registry of Lobbyists, 2023; Schreiber, 2024) and made early patient referrals to MAiD providers after legalization in 2016 (Green, 2022a). Its current advisors include members of CAMAP (CAMAP, 2022a; Dobec, 2021).

Seguin also strongly supported 12-year-old Tracy Latimer’s (who had a disability) father and murderer, whom she claimed received a “quite unconscionable” prison term because he had “lived under a sentence” by her existence (Farnsworth, 1994; Peters, 2021). Together, these comments raise the question of whether any of the illicit deaths involving or known to DWDC were at the request of those caregivers who contacted them and involuntary to the deceased person—including children. Before MAiD, DWDC also hosted both a newsletter and speakers promoting methods of assisted suicide and “self-deliverance” (Dienesch, 2003; Dying with Dignity, 2000). Recently, similar activity by a Canadian has resulted in murder and counselling suicide charges and investigations in Canada, the United Kingdom, and potentially elsewhere (Yousif, 2024).

Both Seguin and Sue Rodriguez, who tried to challenge Canada’s assisted dying prohibition in the courts, later died with the help of or by suicide in the presence of unidentified physicians (Mykitiuk & Paltiel, 2014; Vollmar, 1999). More recently, a journalist on social media reported that the “President of the Canadian Humanist Society,” present at the MAiD death of Rosina Kamis in 2021, stated that “this was ‘only his first legal MAiD’” as “[he] worked with Dying with Dignity Canada for over a decade as a ‘client support advisor’… and helped ‘many’ people ‘die in dignity’ before 2016” (Raikin, 2023a). Elsewhere, a bioethicist favouring an “autonomy-only” rationale for adult MAiD and disabled newborns with parental consent (Davis & Mathison, 2020; Mathison, 2022) states that he has “witnessed multiple assisted deaths and…done many more debriefs with MAID providers” (Mathison, 2024). These comments highlight the possible continuing presence or involvement of non-statutory ideological or sectarian third parties in deaths up to the legal EAS/MAiD era.

In another instance, American physician Georges Reding (a colleague of convicted murderer and pro-euthanasia activist ‘Jack’ Kevorkian), who absconded from his own murder trial (*State of New Mexico vs. Georges Reding*, 2009), was reportedly involved in Canadian deaths in the 1990s (Associated Press, 1999). While some attempted prosecutions of clinicians from this era are known (e.g., *R. v. Morrison*, 1998), they involve single deaths with mixed success, and none seem to include those associated with DWDC. Legal experts at the time reasoned that such deaths “are not prosecuted in a consistent manner because of the potential difficulties in securing a conviction. This may result, with respect to the same factual situation, in no charges being laid or in charges ranging from administering a noxious substance to first degree murder” (Special Senate Committee on Euthanasia & Assisted Suicide, 1995).

Like historical instances, present-day prosecutions (successful or not) tend to involve private citizens, armed forces members, or clinicians involved in homicide and suicide aid but with no or unknown affiliations with pro-EAS organizations (Benedet & Grant, 2023; Daigle & Stunt, 2024; Frank, 2020; *R. v. Latimer*, 2001; *R. v. Morrison*, 1998; *R. v. Semrau*, 2010).

These admissions and news reports suggest Canada has a decades-long pre-MAiD history of unprosecuted involvement in homicide and suicide assistance by supporters of assisted dying and euthanasia and their organizations.

## Healthcare Serial Killing

Miller (2014a) synthesizes earlier work to categorize SK as custodial, delusional, utilitarian, or sadistic in their motivation (Table 1). These are not exclusive categories and can overlap, with HSK typically falling under, but not limited to the “custodial” label. Others identify a “mercy-hero” HSK who acts out of misplaced compassion and lacks elements of the pleasure-seeking cruelty of sadism (Soria & Ansa, 2016).

**Table 1:** Types of serial killers

Serial murderer types (Miller, 2014a, p. 6)	Description
Custodial	“Murder helpless or dependent persons under their care”
Delusional	“On a mission…psychotic or more ideologically-driven, to rid the world of persons they consider undesirable”
Utilitarian	“Whose motive at least partly involves some practical financial or other material gain, although the motive may be mixed with anger or revenge”
Sexual/sadistic	“Kill for the intense pleasure derived from the domination, control, torture, humiliation, and murder of another human being”
Mercy-hero (Soria & Ansa, 2016, p. 19)	Kills out of “compassion feelings for the victims”

Assessing the Canadian MAID system against such features allows us to determine the adequacy of its safeguards to protect patients from clinicians who may share motivations with SKs and HSKs (Haggerty, 2009). First, it is helpful to outline some relevant and well-known cases of convicted healthcare serial murderers (Wettlaufer, Cullen, Shipman, and Letby) and unprosecuted killings or misused end-of-life care (Gosport War Memorial Hospital, Liverpool Care Pathway) that showcase key HSK features (Menshawey & Menshawey, 2022; Miller, 2014a, 2014b; Tang, 2020; Tilley et al., 2019; Yardley & Wilson, 2016; Yorker et al., 2006).

### Wettlaufer: Confidentiality, Lack of Vetting, Anger Issues, Euphoria

Canadian Elizabeth Wettlaufer is a former long-term care nurse and custodial murderer who was only caught through her confession (Foong-Reichert et al., 2021; Frank, 2020; Gillese, 2019; Tang, 2020; Tilley et al., 2019). She murdered eight care home residents and tried to kill others with lethal insulin injections out of anger and frustration with her job and life, and felt euphoric doing so, claiming her victims were sexual harassers or begged for death (Gillese, 2019; Tang, 2020; Tilley et al., 2019). She stole opioids, was subject to complaints, suspended from work, and nearly dismissed. However, confidentiality agreements prevented new employers from learning of her history, allowing her to murder a further resident. Wettlaufer is cited as an example of how Canada’s dated model of provincially fragmented and self-regulated healthcare delivery and oversight inhibits identifying and stopping criminal and incompetent clinicians (Foong-Reichert et al., 2021).

### Shipman: Deception, Falsifying Records, Lack of Vetting, Disbelief by Colleagues

Harold Shipman, a deceased former British medical doctor and custodial killer, murdered hundreds of patients through lethal injections of stockpiled diamorphine. He escaped detection for decades, often by falsifying victims’ death certificates and medical notes (Esmail, 2005; Jackson & Smith, 2004; Kinnell, 2000; Smith, 2002, 2003a, 2003b). Despite a long history of addictions and suspensions, he was popular and allowed to continue practising medicine. Clinicians who noticed anomalies disbelieved and failed to report them (Dyer, 2005). Shipman was finally caught following complaints to authorities by a clinician and a taxi driver who flagged his high death rates. It is speculated that Shipman, like other serial killers (Lankford & Hayes, 2022), was a sociopath who found killing addictive (Diamandis et al., 2023; Esmail, 2005).

### Cullen: Relieving Suffering, Lack of Vetting, Lack of Capacity to Consent, Gratification

Charles Cullen is a former US nurse and custodial killer imprisoned for murdering 29 elderly or seriously ill patients, and potentially hundreds more, who lacked capacity with digoxin and insulin at medical facilities (Hawkins, 2022; Simons, 2022). He had a troubled childhood, military career, marriage and a pattern of mental health issues, suicide attempts, and disturbing behaviour. Despite numerous complaints and other red flags, he escaped detection by changing employers, indecisive managers, lack of safe co-worker reporting and information-sharing between facilities, and failures to report him to police. Cullen claimed he “felt very compelled, very driven, to end suffering as I saw it. I found myself…feeling like I couldn’t watch people hurt, die, be treated like nonhumans” (Hawkins, 2022) and that he “thought people weren’t suffering anymore” and believed he “was helping” (Simons, 2022).

### Letby: Managers Dismiss Warnings

Former nurse Lucy Letby murdered seven infants in a UK neonatal ward from 2015 to 2016 and attempted to kill six others (Dyer, 2023b). Consultants who alerted hospital management of the unusual deaths were dismissed and were forced to apologize to Letby, who had accused them of bullying. A review of Letby’s deaths identified several for further investigation, but this was not followed up and was treated as an “exoneration” by hospital managers (Dyer, 2023b). Letby was only caught after consultants continued campaigning for a police investigation. In October 2023, police announced a corporate manslaughter investigation into the hospital that employed her (Hirst & Lazaro, 2023).

### Gosport War Memorial Hospital: Conceptual Confusion, Euphemisms, Priorities

An inquiry and report at the Gosport War Memorial Hospital in the UK were prompted by two decades of ignored and dismissed complaints from families and staff about the care and deaths of patients between 1987 and 2001 (Jones et al., 2018). The inquiry found that Dr. Jane Barton ordered inappropriate applications of diamorphine by syringe driver, causing the premature deaths of patients, under a personal and organizational approach that prioritized palliation over recovery (Crofts, 2022; Jones et al., 2018). Substantial action to stop the homicides only occurred after they were termed “unlawful killing” instead of describing the killings “in terms of health discourse rather than criminal legal discourse” or using euphemistic language like “make comfortable” (Crofts, 2022, pp. 158, 160, 162). While the inquiry acknowledged homicide (Jones et al., 2018), criminal prosecution did not initially proceed due to a lower likelihood of success. However, in 2023, police announced they had 19 suspects, 22 years after the last death (Dyer, 2023a), indicating the difficulty HSK investigations face.

### Liverpool Care Pathway: Checklist Approach, Poor Leadership, Hastened Death

While not strictly serial homicide, the *Liverpool Care Pathway* (LCP) hospice-level, end-of-life care management approach was upscaled for wider hospital use (Neuberger, 2013, 2016). It was found to be amenable to a blind checklist approach to care and misinterpretation as “a way of deliberately hastening death” (Neuberger, 2013, p. 33). A formal inquiry, report, and abandonment of the LCP followed (Neuberger, 2013, 2016). The review cited poor leadership, insufficient training in the LCP, lack of experience or training in the clinical features of dying, and some clinicians’ negative attitudes toward older patients as factors prompting misuse, and criticized the incentive payments some clinicians received for applying it (Neuberger, 2013). It is relevant to MAiD as a lesson in the dangers of employing an inconsistently understood and applied end-of-life scheme to the same patient cohorts fitting MAiD eligibility and targeted by HSK.

## Healthcare Serial Killing and MAiD

Past recognition of criminal homicides in medical settings has been obscured by euphemistic language and challenges with the prosecution of identified offences (Crofts, 2022; Special Senate Committee on Euthanasia & Assisted Suicide, 1995). However, *killing* is the action and result of EAS, which in MAID’s euthanasia norm is the intentional taking of a person’s life by a physician or nurse practitioner through the injection or provision of lethal doses of common medical substances (Criminal Code, 1985; Pullman, 2023a; Sneiderman, 1994), regardless of culpability or prosecution status.

### Defining Culpable HSK in MAiD

While some patients may be legally eligible for MAiD by meeting the Criminal Code eligibility criteria, their assessors and providers can still have non-clinical or extra-legal motivations to participate, such as sadism, financial gain, misapplied altruism, or ideology (Lyon, 2024). MAiD clinicians might, therefore, commit individual or serial *culpable homicide* (murder, manslaughter, etc.) when they (1) approve and kill objectively MAiD-ineligible people; (2) conduct, coerce, or otherwise influence an assessment, interpret eligibility criteria (e.g., to favour approval), or interact with a person in such a way that the person chooses, is more likely to choose, or is non- or involuntarily compelled, toward death; or (3) kill those persons who may be technically MAiD-eligible but employ non-clinical motives and rationales (Lyon, 2024). Point (3) is a crime absent in Canadian MAiD law but present in Swiss law, which criminalizes “selfish” assisted suicide (Downie et al., 2022; Swiss Criminal Code, 1937, art. 115)^5^ and alluded to under UK prosecutorial guidance for illegal “mercy killings” that are “not wholly motivated by compassion” (Crown Prosecution Service, 2023). Readers might also consider the prospect of a fourth criterion, where MAiD is approved or provided to someone before accessing adequate “palliative, psychological, rehabilitative and social support,” which some clinicians consider to be a medical error (Gallagher et al., 2020; Koch, 2022, p. 366). However, unlike non-Canadian jurisdictions, such access and service provisions are not presently required prior to MAiD, which is viewed as a first-line and not a last-resort treatment.

Because MAiD typically involves clinicians who sequentially assess and euthanize two or more patients, it can be accurately described as *non culpable* *serial homicide*, a legal form of HSK, if Criminal Code eligibility criteria and safeguards are met. If clinicians do not adhere to these safeguards and criteria, they commit *culpable* serial homicide.

Whether MAID’s regime may facilitate criminal HSK hinges on concerns about the adequacy of safeguards emerging from academics, clinicians, MAiD practitioners and colleagues, journalists, and family members of MAiD recipients (e.g., Anderssen, 2023; Parliament of Canada, 2022; Gaind et al., 2023; Ho et al., 2021; Koch, 2022; Kotalik, 2023b; Li & Agrba, 2023; Martens, 2023; Raikin, 2022, 2023b). Despite these issues, risks of culpable homicide do not explicitly feature in today’s MAID discourse, beyond limited prospective arguments it might “veil…homicides occurring within the health system—whether consensual or not” (Crofts, 2022, p. 156; Gazette Desk, 2023). Some features of Canada’s MAID system that might support this position are described next, organized by clinician and structural issues.

### Clinician Issues

#### Broad Criminal Exemptions for MAiD Providers

The potential problem of HSK in MAiD begins with the unprecedented Criminal Code exemptions from homicide and suicide offences (Criminal Code, 1985), effectively legalizing a form of HSK through a loophole missing from other HSK instances, in addition to the pre-existing difficulty in prosecuting known cases (Gorman, 1999; Special Senate Committee on Euthanasia & Assisted Suicide, 1995). In this light, MAiD may have rendered prosecutions of clinicians even more difficult or lenient, perhaps evidenced by the lack of charges so far in any of the cases identified as questionable or legally non-compliant MAiD in the news and official reporting (Coelho et al., 2023).

#### Self-Reported Post-Mortem Legal Compliance and Lack of Law Enforcement

Outside Québec, inconsistent MAiD oversight largely fails to track legal non-compliance (Kotalik, 2023b), a function of Canada’s general reliance on clinicians’ self-reporting of errors to provincial regulatory colleges, which may not disclose disciplinary investigations and can exclude police (Foong-Reichert et al., 2021).

Like the LCP, MAiD is amenable to narrow checklists, which may be used post-mortem to determine the lawfulness of a death (Pesut et al., 2020). MAiD nursing staff who used a checklist had difficulty with their local post-mortem, self-reported score sheet, which applies “rules of a complex legislated and reporting process that determines the line between assisted death and murder” (Pesut et al., 2020, p. 10), with “such leeway in interpretation” that “made it difficult for nurses…to feel as though they were fulfilling their obligation to practice within clear and specific rules” (Pesut et al., 2020, p. 11), which was also concerning to other providers (Li & Agrba, 2023).

For instance, Ontario’s oversight system uses a five-level scale to determine how criminal and regulatory violations are dealt with, ranging from informal conversation, notice emails, regulatory college review, and police involvement. Providing MAiD to a person without a mandatory “serious and incurable illness, disease or disability,” a direct violation of criminal law, requires a referral to the “applicable regulatory body” but not necessarily the police (Government of Ontario, 2023a). Other offences, such as failing to adhere to the 90-day Track 2 assessment period, receive only a “notice email” unless three events involve the same clinician (Government of Ontario, 2023a). This way, Ontario MAiD providers seem to be able to unlawfully kill at least three people before serious oversight interventions are considered. Falsified records were a feature of Shipman’s deaths, and the self-reported compliance with MAiD law seems to provide a similar opportunity for MAiD clinicians.

In Quebec, the Commission sur les soins de fin de vie (translation: Commission on End-of-life Care), a singular independent provincial MAiD oversight body, for example, reported that over 2021–2022, 15 deaths were non-compliant with the provincial or federal MAiD law (Commission sur les soins de fin de vie, 2022; Lane, 2023). These cases were sent to Québec’s College of Physicians for review (Lane, 2023), which appears not to have sent them to the police, with its president publicly denouncing the commission’s warnings as “intimidation.” This dismissive response from the College is reminiscent of managers’ responses to concerns about Letby and Shipman’s murders (Dyer, 2023b; Hirst & Lazaro, 2023). Québec’s health minister, however, has called for explanations following these reports and warnings (Carabin, 2023).

Unlike other MAiD jurisdictions, Canada uniquely lacks an oversight system to independently review MAiD requests, consistent post-death reporting, and a waiting period between approval and death (Kono et al., 2023; Worthington et al., 2022a, 2022b). Despite known referrals (Cook, 2023), no police investigations or charges are yet known in these cases of federal criminal and provincial law violations (Kotalik, 2023b). It is also dubious that healthcare systems could readily spot and stop culpable homicides in MAiD by relying on self-reported legal compliance, filtering possible criminal activity through local oversight or regulatory colleges before (electively) involving police, or operating without a supervisory unit at least as empowered as Québec’s MAiD commission.

#### Clinician Power to Independently Define and Apply MAiD

At least one provider is concerned that the “current legislation leaves too much responsibility in the hands of clinicians, whose application of the eligibility criteria according to their values can render the legislative safeguards impotent.” (Christie & Li, 2023b; Parliament of Canada, 2022; Li, 2023). While recently recommended (MAID Practice Standards Task Group, 2023), clinicians are not legally mandated to collect collateral information from other health or social care providers, associates or family, and other sources that may reveal information showing patient ineligibility, unexplored opportunities for care, or misdiagnoses. The recently released optional Model Practice Standard only suggests clinicians find patients ineligible if they refuse to let them access these sources (MAID Practice Standards Task Group, 2023). However, this standard has not yet been formally embedded in provincial practice standards (CPSBC, 2024; CPSM, 2021; NANB, 2023), but some elements appear in draft updates (CPSA, 2023). If clinicians fail to seek collateral information or specialist experience, it may lead to lethal errors. For instance, MAiD was mentioned to a patient later found to be misdiagnosed with a terminal illness where, fortunately, further explorations revealed a treatable condition (Greenslade, 2023). Deaths may already have resulted from uncaught errors, and a recent estimate suggests a range of 2000 to 4000 such mistakes among an estimated 60,000 MAiD deaths as of early 2024 (Schipper, 2024).

However, MAiD clinicians imply that a MAiD approval and death based on inaccurate patient information might not legally or clinically matter, stating, “[t]hat’s been really hard to get into peoples’ heads suffering is the one eligibility criteria of the assisted dying clause that says that the MAID providers don’t assess whether it’s true or not” and that they “cannot stop patients from lying” (Cecco, 2024; Pesut et al., 2021, p. 8). Such views and CAMAP guidance allowing MAiD for suicidal intention (CAMAP, 2022b) might provide MAiD clinicians cover to kill people without pre-existing eligible health conditions (Lyon, 2024).

Providers have also variously claimed they are not “killing patients” (Marshall, 2023) or that they are providing “care,” not “euthanasia” (Paikin, 2022)*.* In other cases, providers have described MAiD deaths as a “very elegant, graceful, dignified” experience (Raikin, 2022) or a “comfortable” process (Woo, 2023) despite uncertain but potentially agonizing complications (Worthington et al., 2022a, 2022b). Whether a MAID death (lethal injection) is a positive experience for recipients is unknowable to those clinicians as there are no survivors to survey (Kirkey, 2022; Lyon, 2024). However, the perception may bias a requestor toward choosing death if they deferentially believe the clinician is an expert or if death is described in appealing terms (Lyon, 2024; Pesut et al., 2024).

Statements from MAiD clinicians also cast MAiD as a positive right, i.e., one that the government must provide and proactively enforce in the constitutional Canadian Charter of Rights and Freedoms (Constitution Act, 1982).

MAiD is a charter right of Canadian citizens. And it seemed to me, with that philosophy…that it was the health authority’s job…to bring MAiD to the patient. (O’Keefe & Favaro, 2022)

We’re now in the process of restoring a Charter right to psychiatric patients. (Szemberg, 2022)

Some legal scholars argue that clinicians who mistakenly believe they are enforcing a positive right with the unrequested presentation of MAiD may edge into coercion or legal precarity if they do so for people “seeking not MAID but rather help for suicidality or other forms of care” (Shariff et al., 2023a, 2023b, p. 2), a concern reflected in some local guidance (Thomas et al., 2023). However, CAMAP’s documents and  an associated legal academic asserts that prosecutors would be uninterested in clinicians who tell ineligible patients about suicidal actions that might qualify for MAiD (CAMAP, 2018; Downie, 2018) and advise medical staff to mention the option of death by MAiD to anyone potentially eligible (Daws et al., 2022).

These sentiments recall the warnings of euphemisms and individual biases in Gosport and LCP and the risks from confusing medical with speculative legal and personal views, allowing clinicians to perceive and shape MAiD and assessments and communication in ways that may favour a death outcome (Lyon, 2024).

#### Financial Incentives

Financial or other material gain is a motive in some serial murders (Gibson, 2010), and the opportunity appears to exist in the compensation structure for MAiD. Some providers are very high-rate, have specialized in expansive forms of MAiD, and have made MAiD their main practice. MAiD, a billable clinical procedure, is perhaps a primary or sole source of their income (Green, 2022a, 2023). A lack of supply of patients may also require actively seeking patients and advertising MAiD services (e.g., Solace, 2024). At least two providers have published widely distributed autobiographies on their MAiD involvement and patient cases, one of which is described as a “bestseller” with their name included on a compensated speakers registry (Green, 2022a; Marmoreo & Schneller, 2022; Simon & Schuster Speakers Bureau, 2024). The Canadian Criminal Code makes it an offence to knowingly receive “financial or other material benefit resulting from that person’s death, other than standard compensation for their services relating to the request” for MAiD (Criminal Code, 1985, sec. 241.2(6)). However, whether this applies to private income or benefits from such books or speaking is uncertain.

#### Mission and Obligation

In keeping with MAiD’s more recent origins in activism and illegal deaths, some providers describe their role in MAiD in mission-oriented terms such as “‘social justice;’ ‘a crusade;’ [and] ‘empowering people’” (Beuthin & Bruce, 2023, p. 5), a Charter right or clinical obligation (Daws et al., 2022; O’Keefe & Favaro, 2022), or an unrestricted vocational mission of “alleviating suffering in any way that I could” (Buchman, n.d.). Another provider comments that they “had to inject” a patient (Beuthin & Bruce, 2023, p. 6), again suggesting a sense of obligation. DWDC’s continued advocacy for further expansions to the mentally ill and children (“mature minors”) (Registry of Lobbyists, 2023), backed by some prominent MAID clinicians, parliamentary panels, and bioethicists suggests that some believe the law is still too restrictive or “wimpy” (Health Canada, 2022a; McPherson et al., 2023; Wiebe, 2019; Wiebe & Mullin, 2023).

Approaching prospective MAiD recipients from a sense of personal duty or desire for social change may confuse a clinical eligibility assessment with a political mission (Lyon, 2024). An account of a provider “counselling” a prospective patient with psychosis to fly to their clinic for MAiD, where an unknown but somehow qualifying physical illness might be found (Anderssen, 2023) and annual reports of numerous deaths that broke federal and provincial laws, including people without a required serious incurable illness or fluctuating eligibility (Commission sur les soins de fin de vie, 2022; 2023), in addition to expansionism, raises the question of whether a mission view can incentivize transgressing laws and safeguards.

#### Sadism and Pleasure

Concerns about the psychological impact of killing on providers are noted (Chabot, 2017), and the Criminal Code does not explicitly prohibit providers from using MAiD for their emotional gratification or to cause emotional harm to witnesses and loved ones (Duncan, 2023; Raikin, 2022; Serota et al., 2023).

Sadism may be associated with non-sexual pleasure derived from opportunities for cruelty toward others and killing or harming sentient creatures (Buckels et al., 2013). Though not always sadistic or sexual, pleasure is also a frequent occurrence of SK (Burris, 2022; Miller, 2014a), may be addictive (Lankford & Hayes, 2022), appears in HSK (Tang, 2020; Yorker et al., 2006), and is a feature of clinical personality disorders (Personality Disorders, 2013). This is important because elation and a sense of liberation from ending suffering are common in HSK (Tang, 2020). MAiD providers likewise describe positive feelings for gratification from killing, including feeling “hyped up on adrenaline,” “very good,” “gratification,” and “satisfaction” (Green, 2022a, pp. 254–256)^6^ or as,

Loving;” “a solitary practice;” “intimate contact;” “heartwarming,” “the most important medicine I do;” “satisfying medicine;” and “rewarding.”…“an ultimate act of compassion; “an honor;” “incredible gift;” “liberating;” “unlike anything I’ve ever…” and “extraordinary work”…“the right thing in the right circumstance.” (Beuthin & Bruce, 2023, p. 5)

In Australia, one provider mentions “a pressing need for sex” after a death (Simons, 2013).

MAiD (as a suicide or homicide) provides opportunities for sadists to cause death to the patient and perhaps cause indirect harm if the provider is aware of a patient using it to emotionally harm people that they dislike (e.g., ex-partners, by scheduling death on their birthdays or weddings) (Raikin, 2022). Providers sometimes appear hostile toward witnesses or family members (Raikin, 2023c; Wiebe, 2019).

Unlike the high risk two-way nature of idealized military and police lethality, SK occurs in one-way situations where killers minimize risks to themselves and maximize the risk of death to victims for their gratification (Haggerty, 2009). MAiD achieves this through low-risk, legally protected homicide, from which some providers gain significant pleasure (Beuthin & Bruce, 2023; Green, 2022a). As mentioned, prosecutions have failed to follow seemingly obvious law violations. By contrast, in the Netherlands, even the failed prosecution of a clinician had a limiting effect on the fulfilment of MAiD requests (Asscher & Van De Vathorst, 2020), suggesting that the visible enforcement of safeguards is essential.

#### Pathological Altruism and Mercy-Heroism

Noted elsewhere (Lyon, 2024) is the “mercy-hero” variation of HSK, which also results from a distorted sense of self and compassion without the power and control features of sadism (Soria & Ansa, 2016). Providers who describe MAiD as “care,” “loving,” “compassion,” or “alleviating suffering,” or as a form of compassion derived from “suffering in someone else and the desire to change that to help them” (Patel, 2023) may align with this possibly pathologically altruistic type of killing (Oakley, 2013).

#### Lack of Clinician Vetting

Public and police inquiries into Wettlaufer, Cullen, Shipman, and Letby found that a lack of supervision, monitoring, or dismissals of concerns enabled continual offending. Red flags in personal histories, complaint and sanction records, and other issues were documented but overlooked because they were rarely collated. Despite recent calls (Lyon, 2024), there is no separate professional vetting or ongoing monitoring system for current or prospective MAiD clinicians. Variation between health authorities, ministries, and regulatory colleges means they may have an inconsistent willingness to track and respond to issues raised by patients, witnesses, family members, and staff, compounded by self-reported compliance (Pesut et al., 2020). These factors can make it difficult to identify problems and remove problematic clinicians (Foong-Reichert et al., 2021).

#### Neutralization

Neutralization is a strategy employed by serial killers, génocidaires, and others who commit homicidal or other harmful acts to justify or disown responsibility for their actions (Alvarez, 1997; Bryant et al., 2018; Kaptein & van Helvoort, 2019; Pettigrew, 2020; Sykes & Matza, 1957; Tang, 2020). A range of techniques are described in this literature, including denying responsibility for injuries and the existence or humanity of victims, condemning critics, appealing to higher loyalties, and distorting facts. Comments from some MAiD providers and advocates are suggestive of neutralization techniques. Table 2 compares an inexhaustive sample of neutralization techniques paired with statements from some clinicians and advocates to show how they are used in the context of MAiD.

**Table 2:** Neutralization techniques used by HSK and MAiD providers’ statements (adapted from Alvarez, 1997; Bryant et al., 2018; Kaptein & van Helvoort, 2019; Minor, 1981; Pettigrew, 2020; Sykes & Matza, 1957; Tang, 2020)

Neutralization technique	Prospective MAiD examples
Denial of responsibility Not responsible for one’s actions	“I don’t believe I’m killing patients. I believe the illness and disease are killing the patients. I don’t use the word killing” (Green, 2022a) “I ask a lot of questions, but I tend to trust my patients…until I’m proven otherwise, I assume they’re telling me the truth.” (Cecco, 2024)
Denial of victim Victim at fault or no victim	Patient is “hoping for an affirmation of their wish to end this life” (Beuthin & Bruce, 2023) “I am facilitating their final wish” (Sakur, 2023) “I assume they're telling me the truth” (Cecco, 2024)
Denial of injury Actions are unharmful	Death is dignity, euthanasia is “elegant, graceful, dignified” or “comfortable” (Raikin, 2022; Woo, 2023) “Alleviate suffering” (Buchman, n.d.) “Reduce suffering” (Marshall, 2023) On harm/trauma to witnesses: “the general perception of these deaths is not a traumatic one…We can’t do a trial obviously so there’s no randomized study to prove anything,” (Downar, 2023) “Contrary to the way most standard hospital deaths go, assisted deaths tend to be closer to joyful. People sing songs. They give speeches.” (Mathison, 2024) “I provide care” (Paikin, 2022) “…A good death by their standards” (The Canadian Press, 2016) “I don’t think I’m harming anybody…I am there to help them at a time of great need” (Sakur, 2023) “One patient said to me just before I had to inject him, he said, ‘thank you for saving my life.’” (Beuthin & Bruce, 2023, p. 6)
Condemn the condemners Opponents lack credibility	“We would not have got this law without the court case [Carter], our politicians were definitely too wimpy to do this. but they were forced to, so they added safeguards, which is what you’d expect out of a politician” (Wiebe, 2019) “It is not the first time I disagree with something the Pope said. I don’t take my directions from him.” (Sakur, 2023)
Appeal to higher loyalties Personally privilege one or some authorities, standards, or guidelines over others	“Doctors don’t usually practice medicine by criminal law, we care a whole lot about the people who licence us, which is my case is this College^a^… and our professional associations who also set standards and guidelines, and for us this is [CAMAP]” (Wiebe, 2019) “MAiD is a Charter right of Canadian citizens” (O’Keefe & Favaro, 2022) “How about we let the patient decide?”(Sakur, 2023)
Defence of necessity Death by MAiD is required	“Alleviate suffering” (Buchman, n.d.) “Reduce suffering” (Marshall, 2023) “…A good death by their standards” (The Canadian Press, 2016) “I am there to help them at a time of great need*”* (Sakur, 2023) “…I had to inject him…” (Beuthin & Bruce, 2023, p. 6)
Denial of humanity Demote the personhood of the victim	MAiD as a provider-centred “experience…of the existential and provides a way to get closer to the unsayable profoundness that occurs in that space of providing death for a suffering other.” (Beuthin & Bruce, 2023)
Distort the facts Misrepresent objective facts and evidence	“I know some people use assisted suicide and euthanasia. My feeling is that I provide care.” (Paikin, 2022) “I don’t believe I’m killing patients…” (Marshall, 2023) MAiD is a “Charter right” (O’Keefe & Favaro, 2022)^b^ Death is dignity, euthanasia is “elegant, graceful, dignified” or “comfortable” (Raikin, 2022; Woo, 2023) “We should be very concerned, if we have reasons to believe that there are cases of Maid that are happening where people don’t fulfil the criteria…that would obviously be completely unacceptable. But we’re not seeing that reflected anywhere.” (Cecco, 2024)^c^ “In reading his paper, one gets the feeling that Lyon has never seen an assisted death…Contrary to the way most standard hospital deaths go, assisted deaths tend to be closer to joyful…” (Mathison, 2024)^d^ “‘I’m from the MAiD team.' In truth, there was no MAiD team: It was a fictitious club that I made up on the spot to try to sound more official” (Green, 2022a, p. 55)

^a^British Columbia College of Physicians and Surgeons

^b^Courts have only ruled on MAiD’s constitutional permissibility relative to the specifics of certain cases, not that it is a positive constitutional right. Legal scholars point out that legislation or practices may extend beyond the limits of the court rulings (Grant, 2023; Shariff, Ross et al., 2023)

^c^Legally non-compliant MAiD deaths are annually documented in Québec, and have been noted in Ontario, British Columbia, and prisons (Coelho et al., 2023; Cook, 2023; Commission sur les soins de fin de vie, 2022;  2023)

^d^This source corrected their original assertion about not witnessing an assisted death when requested by Lyon

### Structural Issues

#### Inconsistent Guidance Across Regions and Between Organizations

Local and regional health authorities differ and may be opposed in their guidance (Thomas et al., 2023). For instance, some permit or obligate clinicians (including non-assessors and providers) to raise MAiD with patients, while others forbid this and require the patient to initiate the conversation or allow family members to kill the patient (Thomas et al., 2023). Clinicians also differ in their perception of sources of guidance, giving varying weight to the Criminal Code, local and regional or federal guidance, CAMAP, or DWDC in forming their approach (Close et al., 2023; Wiebe, 2019), and some informal approaches in use may be contrary to the law and official guidance (WV v MV, 2024, detailed later). These issues reinforce concerns about bias voiced by other providers (Li, 2023), confirming the challenges of policing MAiD (Lyon, 2024; Muldoon et al., 1982).

#### Bureaucratic Obstacles and Pre- and Post-Mortem Privacy Law

In Gosport, family members were among those raising concerns but were “marginalised” by healthcare staff (Jones et al., 2018), while in Letby, managers dismissed concerns. In MAiD, such obstacles are compounded by patient privacy laws and the onus on family members to flag issues.

Reports suggest that individual attempts to have MAiD cases reviewed face bureaucratic hurdles. Health authorities may block family and police requests to access medical or Criminal Code criteria may be too broad to be useful when there is official doubt about the legitimacy of a MAiD death (Anderssen, 2023; Parliament of Canada, 2022; Serota et al., 2023). Regulatory colleges have dismissed complaints against providers and declined police referrals for questionable MAiD practices and deaths (Esler, 2018; Gentile & Bolly, 2023; Lane, 1994; Rose, 2019; Wilson, 2018).

Canadian privacy laws also maintain patients’ post-mortem privacy rights regarding health records. Generally, however, exceptions are made for the deceased’s legal representatives, such as estate executors (Canadian Medical Protective Association, n.d.), but this is not always the case. In British Columbia, for example, gatekeepers determine whether the request is in the “best interest of the deceased and whether the disclosure would be an unreasonable invasion of the deceased’s privacy” (Interior Health, 2023). Applicants can appeal a refusal to the provincial privacy commissioner, but only within 30 days of the date of refusal, which may be issued by the postal service and further narrow the appeal window.

In a premortem case brought before an Alberta court, a father (WV) attempted to have a court review his 27-year-old daughter’s (MV) MAiD approval and uphold an injunction pausing her access, given that she had no known eligible physical health condition, though she had ineligible mental illnesses. The initial ruling (pending appeal) found WV had no standing “private medical decisions or the clinical judgment of her doctors,” which could not be reviewed by a court (WV v MV, 2024), allowing MAiD to proceed pending the outcome of an appeal. Though it did not affect MV’s access to MAiD, the judge suggested that the health authority responsible for selecting assessors may be legally liable for its unofficial use of a “tiebreaker” assessor who had found the patient eligible during earlier rounds of assessments (via assessor shopping), which did not result in the necessary two approvals. The judge also allowed WV a limited public standing given the “importance of resolving these issues for MAiD practitioners and people interested in applying for MAiD” (WV v MV, 2024).

Nonetheless, a former justice minister responsible for MAiD stated that “Canadians” (i.e., patients’ family members, if they exist) must provide oversight (Coelho et al., 2023; The Fifth Estate, 2023). Yet, patients’ family members and associates have very limited legal standing in MAiD, making it especially difficult to justify access to assessment records yet facile to find grounds to refuse such access. Thus, these documents and the clinicians who create them are shielded from legal scrutiny.

#### Imprecise or Poorly Defined Concepts

The Wettlaufer inquiry also included recommendations for death reporting, training, and clear definitions of key terms like “reasonable grounds” and “improper or incompetent treatment” in policies (Gillese, 2019, p. 27). Likewise, in Gosport and the LCP, conceptual imprecision and misunderstanding confused homicide with healthcare (Crofts, 2022). In MAID, the Criminal Code does not define its critical terms like “irremediable,” “intolerable,” “suffering,” and “reasonably foreseeable,” leaving those to be subjectively applied by clinicians or the patient (Centre for Clinical Ethics, 2023; Christie & Li, 2023a; Lyon, 2024; Shour & Li, 2021; Sibbald, 2016). This confusion may make it challenging to identify a clinician’s intention to determine whether a death is criminal, a “reasonable mistake”, or within the law (Crofts, 2022; Keown, 2018). As noted elsewhere (Lyon, 2024), in a CAMAP exchange discussing completing suicide attempts with MAiD, a provider worries that imprecision means an “anti-MAiD prosecutor” or coroner would apply a legal understanding of natural death that differed from their subjective views, resulting in a “court case” that might distress the provider—with no mention of concerns about preventing a lethal medical error or distress to their loved ones or witnesses (Raikin, 2023b). Other materials, however, suggest that interpreting the core eligibility criteria of “reasonably foreseeable” and “natural death” allows assessors to mention suicidal intentions and actions to ineligible patients that they could undertake to become eligible and that this would not be prosecuted (CAMAP, 2018, 2022b; Downie, 2018). Poorly defined legal language, thus, may allow MAiD clinicians to control the process and outcome of an MAiD assessment in ways that might typically be criminal (Lyon, 2024).

#### Hastening Death

A feature of abuses in the hospitals that employed the LCP was the misunderstanding or misapplication of a clinical framework as a process to hasten death. Hastening death is also an argument used both for and against MAiD, which is framed as a charitable act to relieve suffering through death or to shortcut deficient social or healthcare systems that cannot provide timely care or social service support (Ashe, 2021; Gallagher et al., 2020; Gallagher & Passmore, 2021; Wiebe & Mullin, 2023). Some providers and advocates claim that patients are held “hostage” to the delays or deficiencies in social welfare and healthcare systems and accordingly justify MAiD as a faster route to end suffering (Downie, 2023; Downie & Schuklenk, 2021; Sakur, 2023). If providers believe that they are *saving* people from underfunded healthcare systems by killing them, it may incentivize further killing. It also creates a variation of the hostage analogy where (implicit threats of) increasing patient MAiD deaths are used to extort improved services from governments.

#### Assessor Shopping, Pairing, and Soliciting

SK and HSK often seek their victims. In MAiD, “assessor shopping” is when patients, deemed ineligible by initial assessors, may eventually find or be sought by two less scrupulous assessors willing to approve and provide MAiD (Coelho et al., 2023; Tang et al., 2023). While discouraged in Québec (Vivre dans la dignité, 2023), a recent study shows that many local and regional health authorities host “policies described ways in which patients could seek additional assessments if the original assessment deemed them ineligible” (Thomas et al., 2023, p. 6). The informal practice of using a “tie-breaker” assessor with a known bias favouring eligibility highlights the danger of assessor shopping, increasing the chance of death (WV v MV, 2024).

Some assessors and providers work in teams rather than independently (e.g., with shackled and guarded prisoners) (Driftmier & Shaw, 2021). Health Canada does not presently track approval-shopping (but may have this capacity) (MAID Statistics, 2023). Complicating this, findings of ineligibility may not be shared between assessors, and patients may not disclose previously failed assessments to new assessors (MAID Statistics, 2023). Amid recommendations to mention MAiD (Daws et al., 2022), assertions that providers will not be prosecuted for mentioning ways the ineligible might self-harm themselves into eligibility (Downie, 2018; Lyon, 2024), at least one provider now advertises their MAiD provision, book, and speaking services personal websites including a copyrighted website called *Solace* (Green, 2024; Solace, 2024). Concerns have also been raised that subjectively presenting the quality of a MAiD death as positive experience, (e.g., as elegant, comfortable, beautiful, or dignity) may coercively violate informed consent standards (Lyon, 2024).

In these ways, clinicians can actively seek opportunities to provide MAiD in ways that may compromise the independence of assessments and increase the likelihood that ineligible, marginal, or uncertain requestors intersect with providers who are more willing to find ways to approve and kill them.

#### Data Deficient and Ambiguous Official Reporting and Recording of MAiD Deaths

Determining what, where, and how deaths are reported varies between jurisdictions (Pesut et al., 2020). Inconsistent data reporting standards can hide patterns and anomalies, such as unusual or unexplained clusters of deaths around specific medical staff, and have inhibited the detection of serial murder (e.g., Cullen, Wettlaufer). Health Canada’s annual MAiD reports, released from 2019 (Health Canada, 2020, 2021, 2022c, 2023), are criticized for deficiencies and omissions that may conceal Criminal Code non-compliance (Kotalik, 2020). Key demographic and other data are only federally reported as of 2023 for inclusion in the 2024 annual report (Health Canada, 2022b). Reporting to provincial coroners varies by province and territory, depending on the structure of their healthcare systems (Kotalik, 2023b). For example, Ontario currently requires that all MAiD deaths be reported to and reviewed by the provincial coroner’s office (Ministry of Health & Long-Term Care, 2023) amid self-reported compliance.^7^ In contrast, British Columbia requires reporting only MAiD deaths that occur in connection to accident, violence, self-inflicted injury, or while in custody (British Columbia Coroner Service, n.d.). Similar issues make identifying and stopping HSK difficult (e.g., Wettlaufer, Cullen).

#### Unmonitored, Unassessed, or Undermined Capacity to Consent to Death

Cullen, Wettlaufer, and Letby took advantage of their victims’ lack of capacity. The Criminal Code permits MAiD if a person loses the capacity to consent through signed waivers (Criminal Code, 1985). However, there is no explicit requirement under MAiD legislation to initially assess or continually monitor consent capacity, though, patients must have the capacity to consent to MAiD. If capacity is only assumed, then it is difficult to know if patients die by MAiD lacking capacity, despite evidence of this occurring in Québec (Commission sur les soins de fin de vie, 2022). Further evidence suggests capacity assessments using recognized clinical tests and qualified staff are rarely employed with some MAiD assessors relying on their “gut” or “intuition” instead (Wiebe et al., 2021). Others discuss or admit to the “covert” sedation of patients to *overcome* their capacity to express distress, which may be a refusal of MAiD, and the need to “calm” witnesses (Raikin, 2023c). Provinces, however, largely leave it to the assessor to determine if capacity should be assessed (CPSBC, 2024), and they are called for by others as suggested in the Model Practice Standard (MAID Practice Standards Task Group, 2023; Selby et al., 2020). A failure to undertake capacity assessments and prosecute providers who kill patients lacking capacity may create a permissive culture and an opportunity for HSK.

### Summary

The issues that enable general HSK provide an initial list of its features and characteristics, which may be used to help develop oversight protocols and safeguards for overlapping possibilities in MAiD. The accompanying supplementary information summarizes these issues in a table and compares them to indicators across SK and HSK.

## Example Providers

Profiles of two public-figure MAiD providers, drawing on their statements and information in publicly available recordings, documents, books, and reporting, illustrate how the Canadian MAiD system may tolerate similarities with HSK. These descriptions do not accuse them of criminal HSK or suggest that it is occurring but serve to illustrate apparent thematic parallels in legal MAiD.

### Dr. Ellen Wiebe

Dr. Wiebe’s primary practice is women’s health and MAiD. She is also a clinical professor at the University of British Columbia (PWIAS, 2023), author of numerous MAiD and other medical journal articles, and a member of DWDC’s Clinician’s Advisory Council (Dying with Dignity Canada, n.d.), CAMAP’s Board of Directors (CAMAP, 2022a), and the Hemlock Aid (2020) organization that has advised people on EAS. By May 2022, she had euthanized at least 430 people (Coelho et al., 2023), or about 1.3% of the 31,664 MAiD deaths by the end of 2021 (Health Canada, 2022c). She is reported to have killed at least one person deemed ineligible for MAiD by other assessors after they travelled to her clinic (Raikin, 2022) and has described her approach to MAiD in mission-oriented terms: “My job is to help them have a good life and a good death by their standards, not by mine or anybody else’s” (The Canadian Press, 2016).

In 2017, Wiebe euthanized a resident of the Louis Brier Home and Hospital against the wishes of the management (Lazaruk, 2018). This facility banned MAiD on its premises as it was home to Holocaust survivors likely to be deeply troubled by nearby euthanasia as it featured in that Nazi atrocity, which shares earlier roots with MAiD (Brody & Cooper, 2014; Grodin et al., 2018). Managers accused her of “sneaking in and killing someone” as she did not inform the facility (Lazaruk, 2018). The College of Physicians and Surgeons of British Columbia (CPSBC) investigated, finding that her actions “did not meet their charging standard” (Rose, 2019). Wiebe neutralized her actions by appealing to authority, claiming that her “highest responsibility” was to her patient and that the facility was the patient’s home (Rose, 2019). She has similarly admitted to entering a Catholic hospital “against the rules” (Slobodian, 2023). In 2018, she was again subject to a complaint to the CPSBC, this time by the Chief Medical Officer of the British Columbia Coroners Service, over the death of someone who did not appear to meet the eligibility requirements for MAiD (Esler, 2018). An investigation found that the requirements were met once the patient began to VSED (Esler, 2018). She has mentioned at least one other eligibility complaint lodged by a patient’s family (Wiebe, 2019).

Wiebe is recorded laughing while participating in a CAMAP discussion on the merits of surreptitiously lowering patients’ capacity to refuse MAiD (Raikin, 2023c), when describing “family members” as “our greatest risk” (Raikin, 2022; Wiebe, 2019), and in other instances where she describes deaths or resistance to her efforts (Slobodian, 2023).^8^ Condemning the condemner, she once called politicians “wimpy” for legislating safeguards and appears to hold patient, CAMAP, and provincial guidance as higher authorities than the Criminal Code (Wiebe, 2019). She has complained about the onerousness of the paperwork required to adhere to the law (Slobodian, 2023).

Wiebe’s public history of MAiD has themes of HSK in that it is custodial, mission-driven, and utilitarian, with possibly sadistic elements in her apparent enjoyment of patient deaths and dismissing, disparaging, subverting, or overcoming patients, loved ones, regulators, organizations, and law that she disagrees with or who resists her efforts. Like HSK, she has a track record of multiple serious formal complaints involving patient deaths against her that, while dismissed, still merited high-level regulatory review.

### Dr. Stefanie Green

With at least 300 deaths (Reyes, 2023), Dr. Green is the “Founding President of the Canadian Association of MAiD Assessors and Providers, a Medical Advisor to the BC Ministry of Health MAiD Oversight Committee, Co-Lead of the Canadian MAiD Curriculum Project” (Green, 2023). She officially “began working almost exclusively in assisted dying” in 2016 despite claiming no experience or training in it beyond attending a three-day euthanasia conference (Green, 2022a; The Fifth Estate, 2023). Later, she published an “internationally bestselling” book about her involvement in MAiD (Green, 2022a, 2023; Simon & Schuster Speakers Bureau, 2024). Describing herself as “an international speaker and educator”, she promotes her publicly-funded MAiD provision and private speaking, book, and other activities on personal websites (Green, 2023, 2024; Solace, 2024). She claims to have “no personal or professional stake” in a potential extension of MAiD to people with mental illness (Green, 2023), despite financially benefitting from MAiD through provision, self and corporate promotions of her book, potential speaking compensation, and the increasing income opportunities from an enlarged candidate pool that such an expansion provides. She justifies herself as “helping” by euthanizing patients she claims are “hostage” to deficient healthcare systems that cannot deliver timely care (Alberga, 2022; Sakur, 2023).

Green claims she can differentiate between what she terms “active” and “stable” cases of mental illness^9^ in MAiD requestors (Raikin, 2022), despite her lack of psychiatry qualification (Minnings, 2023). She also describes how a turbulent childhood helped her “compartmentalize my life, such that I could do my MAiD work and not be wrecked or overwhelmed by it” and that performing euthanasia stimulates an adrenaline high and a “very good” feeling amid her concern that some might see her “as a psychopath” for this affect (Green, 2022a, pp. 134, 107). Green has distorted facts and denied responsibility by falsely claiming that she was part of a fictional “MAiD team…to try to sound more official” to gain access to a patient (Green, 2022a, p. 55) and that “I don’t believe I’m killing patients” (Marshall, 2023), though she approves and administers lethal injections and track 2 patients cannot die from non-terminal conditions (Lyon, 2024). She regrets not approving patients later approved by others (Green, 2022b). While claiming to be “committed to providing the highest standard of medical care possible under any and all legislation” (Green, 2023), Green’s approach to MAiD shares themes with custodial, utilitarian, and sadistic or mercy-hero HSK descriptions as she financially and emotionally benefits from her homicides, makes self-promotional claims about her status and skill, suggests she is helping, yet also justifies and distances herself from her actions.

## Discussion: Who Shall Watch the Watchers?

While the discussion and profiles here do not accuse or evidence criminally culpable HSK in MAiD, they suggest the system can support disproportionately prolific MAiD providers with readily identifiable thematic similarities. This scenario raises the troubling question of whether it would be possible to recognize criminal HSK in the current system if it tolerates, creates, or draws such personalities amid legal ambiguity and inconsistent guidance and practices. A Shipman, Cullen, Wetlaufer personality might operate undetected as a MAiD provider, either by killing only MAiD-eligible patients for personal benefit or misguided altruism or by leveraging unsupervised control over assessments and records to shape assessments toward an outcome of death or conceal homicides of ineligible people (Lyon, 2024). The possibility that abuses may occur more readily in some regions is exacerbated by wide variations of local guidance and individual approaches to MAiD (Close et al., 2023; Thomas et al., 2023). An obvious systemic safeguard is the requirement for two “independent” assessments and seeking a third, if deemed necessary, with expertise in the patient’s condition. However, this safeguard may be subverted through unlimited and unrecorded “assessor shopping,” unsanctioned use of approval-biased “tie-breaker” assessors (*WV v MV*, 2024), obligatory referrals to (potentially permissive) assessors (Daws et al., 2022), clinicians who pair to assess and provide concurrently (Driftmier & Shaw, 2021), and the perception that relative experience counts as formal expertise (Lametti, 2021; Close et al., 2023).

Courts have ruled on the constitutional status of MAiD, meaning some version(s) of it will likely feature in Canadian society for the foreseeable future (Lemmens, 2023). Assuming MAiD remains legal and cannot be prevented from attracting or producing (Elliott, 2022) homicidally-inclined or benefitting clinicians, it must be made *as safe as possible* to ‘protect patients,’ maintain public trust in healthcare systems and clinicians. The task then is to identify and limit opportunities for abuse by reforming MAiD delivery and instituting more robust oversight, which might include the following recommendations:

### Restructure MAiD Delivery

Restructure how MAiD is delivered to limit the incentives and opportunities for clinicians to mobilize illegitimate reasons to kill patients, e.g.,

#### Remove the Euthanasia Option for MAiD; Only Permit Self-Administration

Removing the euthanasia option removes the hands-on instance of homicide, blunting the ability of clinicians to find personal gratification and financial benefit from the act of homicide. Death rates are as much as 20 times lower in areas where patients must self-administer lethal doses, suggesting this may have merit (Pullman, 2023b).

#### Restructure Assessment and Provision Protocol Away from Just Two Clinicians

Assessments for MAiD could improve upon the team-oriented approach already used in some Canadian jurisdictions (Thomas et al., 2023). Such diluted assessments should include appeal, review, veto, and revocation powers to remove the power of individual clinicians to mobilize non-clinical or criminal reasons to approve and provide death and shape assessments to favour it (Lyon, 2024).

#### Repeal Track 2 MAiD, Legislate More Precise Track 1 Conditions

MAiD for people who are not dying may cloud the medical rationale for approvals, allowing a personally motivated (Lyon, 2024) clinician to exceed safeguards. This can be remedied by repealing track 2 (Grant, 2023). Track 1 safeguards can be clarified and tightened through federal criminal law, and currently underused provincial healthcare and other laws and regulations (Shariff, Ross, et al., 2023). Measures might include statutory requirements for explicit temporal prognoses of deaths and clear definitions of key concepts like “intolerable suffering” to make it harder for clinicians to mobilize bias (Li, 2023; Lyon, 2024).

#### Ban the Promotion of MAiD by Advocacy Organizations and Clinicians, and Set Clear National Standards for All Communication About MAiD

Closely linked pro-MAiD organizations and individual clinicians and allies promote MAiD as a form of choice, dignity, and a solution to suffering. There are also reports that this happens before or during assessments (Raikin, 2022; Woo, 2023), which may be coercive (Lyon, 2024; Shariff & Ross, 2023). Banning the promotion and marketing of MAiD and using nationally standardized scripts that adhere to the informed consent doctrine creates a more neutral environment for patients to interpret the risks and benefits of MAiD on their terms, preserving autonomy (Lyon, 2024).

#### Ban Financial Dependency and Enforce the Criminal Prohibition on Extra Benefits

Limiting the amount of time and income clinicians can spend on and derive from MAiD restricts the incentives to provide MAiD for financial benefit. While Canadian law prohibits clinicians from knowingly benefitting “under the will of the person making the request, or a recipient, in any other way, of a financial or other material benefit resulting from that person’s death, other than standard compensation for their services relating to the request” (Criminal Code, 1985, sec. 241.2(6b)), it currently does not seem to be applied or enforced for private income from book sales or other sources. Changing the model of compensation for clinicians and applying existing law removes incentives to increase death rates.

#### Criminalize ‘Selfish’ Provider Motives for MAID

Clinicians’ affective benefits from euthanasia are more complex incentives to moderate than visible financial or material benefits. However, creating an offence prohibiting clinicians from providing MAiD for selfish motives or profiteering from deaths can disincentivize approvals and deaths for these reasons and give overseers and justice systems a concrete tool to block such clinicians’ involvement in MAiD (e.g., Swiss Criminal Code, 1937, sec. 115).

### Institute a ‘Meta-Regulator’ Oversight System of Canada’s Healthcare System

The Wettlaufer case and other patient safety concerns led some researchers to recommend expanding the overhaul of some of Canada’s self-regulating provincial healthcare systems by creating a federal *meta-regulator* (Downie et al., 2006; Foong-Reichert et al., 2021). A meta-regulator is an “oversight body independent from government to audit regulators, set regulation policies and practices and oversee the appointment of the Board of Directors of each regulatory body” (Foong-Reichert et al., 2021, p. 23). For MAiD, such a meta-regulator might include:  

#### Transparent and Independent Federal Oversight, Review, Information and Data Sharing, and Redress Mechanisms

Canada currently lacks a federal database of “discipline and malpractice claims against professionals” and relies on self-reporting issues, including criminal charges or convictions of physicians moving between provinces (Foong-Reichert et al., 2021). The lack of data-sharing for eligibility issues or assessor-shopping is also an opportunity to conceal abuse. An arm of the meta-regulator, empowered with broad investigative authority, resources, and data sharing, perhaps like Québec’s MAiD commission, but with more explicit enforcement powers and links to police and prosecution services, will allow the review of local and nationally aggregated MAiD data, simplifying the investigation of violations, anomalies, or concerns. Further steps may include creating exemptions to privacy legislation for MAiD deaths, as public accounts describe local health authorities using privacy law to block access to police and family or others who question a death. Further recommendations are also found in Kotalik (2023a, 2023b) and Foong-Reichert et al. (2021).

#### Clear Guidance for Police, Prosecution, and Judicial Services to Recognize Offences in MAiD

MAiD law is ambiguous, with many exemptions for clinicians that may make it difficult to lay charges or prosecute apparent offences, a current and historic problem within healthcare EAS (Crofts, 2022; Special Senate Committee on Euthanasia & Assisted Suicide, 1995). Gosport shows this can happen even in obvious healthcare serial killing, and the LCP shows how a misapplied end-of-life practice lacking standardization can lead to errors. Greater clarity and precision in the Criminal Code for MAiD definitions and concepts can help to identify crimes and incompetence, making it easier to locate and remove such clinicians before *more* harm is done (Foong-Reichert et al., 2021). Further measures might be to make assessments available for judicial or other review *before* a death (e.g., WV v MV, 2024). Canada could quickly draw on recommendations and experiences with HSK and lethally deficient care from its own and similar common law and healthcare jurisdictions (Crown Prosecution Service, 2023; Gillese, 2019; Jones et al., 2018; Smith, 2002, 2003a, 2003b).

#### Vetting and Monitoring of MAiD Assessors, Providers, and Associated Clinical Staff Within a Searchable National Database

HSK often have chequered employment histories and psychological or psychiatric issues, including personality disorders, interpersonal issues, and problems like substance abuse. Vetting, a frequent recommendation from serial healthcare murder case reviews (Foong-Reichert et al., 2021; Tilley et al., 2019), and monitoring will help to identify and stop abuses and unsuitable providers (Lyon, 2024). Including practitioners’ records and evaluations in a national database will allow for easier monitoring and prevent the relocations preventing detection in HSK.

#### Replacing CAMAP with a Publicly Accountable National Stakeholder Organization

CAMAP’s power to define clinical MAiD training and knowledge sharing behind closed doors leaves much of its activity beyond public and regulatory scrutiny. Reported materials host concerning comments from its members (Raikin, 2022, 2023b, 2023c) and some of its guidance may be poorly grounded in clinical standards and ruled law (Lyon, 2024). Replacing it with a transparent, independent, public, national organization with equitable and diverse stakeholder membership (e.g., disability and Indigenous communities) that conceives MAiD training and knowledge within the bounds of clear law limits the potential for its members’ biases to influence practice.

### Test the System

Red-teaming and system checks, such as concealed competency assessments, may test its sensitivity to detecting anomalies and offences (Foong-Reichert et al., 2021).

## Limitations

While provocative, this paper is limited to critically assessing the MAiD system as an opportunity structure for culpable HSK to highlight gaps in the current safeguards. It does not assess that criminally culpable homicide (murder, manslaughter, etc.) is happening, only that it could, and that Canada’s MAiD regime may serve as a protectorate that allows serially homicidal personalities to “safely” or legally kill. How society and medicine reconcile with that possibility or other issues of the morality, ethics, or clinical and social benefits or harms of MAiD are beyond consideration here. Additionally, the examples cited in this paper are drawn entirely from publicly available sources. It is unknown what confidential or internal information may be available to confirm or mitigate these concerns—yet it is already easy to identify fundamental problems with the safeguards. This paper also addresses the possibility of malfeasance on the supply, rather than demand, side of MAiD. Further criminological or sociological research might, for example, examine MAiD through a “victim-offender overlap” lens (Berg & Schreck, 2022) and the influence of promotional marketing by DWDC and others. Other parallels may appear between MAiD assessment and provision and the idea of HSK as a confidence trick (Lubaszka et al., 2014), especially where a provider frames MAiD (death) in attractive language to patients and their caregivers (Lyon, 2024). Related work could also explore the possibility, perhaps implicit in some recent reporting, that MAiD requestors may manipulate law and safeguards or individual providers (Cecco, 2024; Raikin, 2023b), akin to “suicide-by-police” (Patton & Fremouw, 2016).

## Conclusion

Canada’s recent history involves unprosecuted admissions of illegal assisted suicide or homicide by clinicians who deemed the law unfair. Now legally protected under MAID, clinicians may sequentially take many lives by euthanasia, committing *serial non-culpable homicide*. It is reasonable to consider that without adequate oversight, some medical practitioners may be drawn to commit MAiD homicides for personal benefit and rationales that diverge from MAiD’s legal, bioethical, and medical justifications (Muldoon et al., 1982), with some suggestion that this could be occurring (Lyon, 2024). Examining the current MAiD system’s ability to enable or conceal serial murder is a valuable way to assess its ability to prevent this occurrence (Crofts, 2022). Such an analysis shows that poor vetting, ambiguous concepts, inconsistent oversight and standards, assessor shopping, and concentrated clinician power and prejudices combine to describe a system bereft of measures to prevent and detect criminally culpable or antisocially motivated individuals operating as MAiD assessors and providers. Remedying these issues will better protect patients and systems from such abuses, which may still have to contend with law-abiding clinicians participating in MAiD for the same benefits and motives as their criminal counterparts—reminding us again of the many warnings of the lethal dangers of bias and poor oversight in EAS (Crofts, 2022; Li, 2023; Lyon, 2024; Muldoon et al., 1982; Schipper, 2020).

## Supplementary Information

Below is the link to the electronic supplementary material.

Supplementary file1 (DOCX 30 KB)

## Footnotes

1 I have personal experience with MAiD, but I have excluded references to this, or anyone involved from this paper.

2 In contrast, the United Kingdom, which is not a MAiD jurisdiction, explicitly guides prosecutors to understand such “mercy killing” as murder when committed by a healthcare professional and to consider withholding prosecution in only highly exceptional (and often controversial e.g., Benedet & Grant, 2023; Koch, 2008) cases involving compassionate motives by relatives (Crown Prosecution Service [CPS], 2023). For example, the CPS (2023) suggests prosecution for murder and related offences where a death was not “wholly motivated by compassion”, someone “influenced the victim not to seek medical treatment, palliative care and/or independent professional advice or denied access to such treatment, care and/or professional support” or was committed by a “healthcare professional” against “a person in their care”. Prosecution is also advised for instances of coercion, where the victim may be “influenced to view themselves as a burden”, was “physically able to undertake the act to end their own life”, or lacked legal decision-making capacity (Crown Prosecution Service, 2023). No similar guidance when to prosecute MAiD related deaths exists. Canadian MAiD, however, is homicide by a healthcare professional against a person in their care, has been provided for suffering from perceived burdensomeness, loneliness, (Health Canada, 2023), and to incapacitated patients (Commission sur les soins de fin de vie, 2022).

3 VSED (voluntary stopping eating and drinking) is a medicalized term for an intentional self-inflicted attempt to die by starvation and dehydration and falls into a self-harming range of remediable “refusal of care” actions, such as not taking essential medication, that may lead to death at some point. While sometimes debated, these actions are suicidal (Birnbacher, 2015; De Leo, 2022; Downie, 2018; Horowitz et al., 2016). Their suicidal nature is uncontested in the original Carter ruling which paved the way for MAiD (*Carter v. Canada* *(Attorney General)*, 2012; Downie, 2018; Lyon, 2024).

4 “Another said that the standard for counselling for suicide is very high in the criminal code and involves active encouragement and proactive steps; speaking in general about how a person could die does not approach the level of counselling for suicide.” (CAMAP, 2018, p. 54).

5 Downie et al. (2022) compares EAS laws across countries and jurisdictions and generally finds the definitions of non-compliance or criminality revolve around clinicians’ adherence to technical procedural and eligibility criteria. Most do not have laws that consider providers’ motives, but those that do (Switzerland, Colombia) make distinctions between mercy killing and culpable homicide around the relative altruistic and selfish motives of the offender. Assisted suicide is legal but euthanasia is a crime in Switzerland, but eligible for a more lenient sentence “for commendable motives, and in particular out of compassion for the victim” (Swiss Criminal Code, 1937, art. 114) whereas someone, including clinicians, “who for selfish motives incites or assists another to commit or attempt” suicide commits an offence (Swiss Criminal Code, 1937, art. 115).

6 E.g., “*I wondered if it was inappropriate to be happy at that moment. A patient I had cared for had just died, after all. I checked in and found myself feeling rather… upbeat, not so much from the music but more like how I felt after delivering a baby. I was startled at this discovery, almost confused. I stopped tapping my toe, turned off the music, and pulled over into a curbside parking spot. Upbeat? Well, yes. I felt like I was a little hyped up on adrenaline. This can’t be right, I thought. But there was no denying it. I did feel good. I felt very good…* “*But it occurred to me that I probably shouldn’t tell anyone. What would they think? ‘I helped someone to die today, and I feel really great about it.’ I might be seen as a psychopath”* (Green, 2022a, p. 231).

7 In practice, “Nurse MAiD Investigators” may be tasked with reviewing cases, not physician coroners (Government of Ontario, 2023b).

8 Grieving or traumatised loved ones or witnesses (Serota et al., 2023), let alone patients, may find such behaviour and affective responses unacceptably inappropriate (Sifferd & Hirstein, 2013).

9 MAiD for sole mental illness is currently culpable homicide in Canada, due to become legal in March 2027 after a parliamentary committee found a lack of preparedness (Arsenault & Martin, 2023). However, MAiD is currently permitted for a person with a mental illness if they meet the criteria for a physical disability (Health Canada, 2024).
