# Supplementary material for: Canada’s Medical Assistance in Dying System can Enable Healthcare Serial Killing
Source: HEC Forum. 2024 Aug 2;37(1):65–105. doi: 10.1007/s10730-024-09528-3 (PMC11832602; doi:10.1007/s10730-024-09528-3)
Supplement: Supplementary file 1 — Supplementary file1 (DOCX 30 KB) [file 10730_2024_9528_MOESM1_ESM.docx]

**Supplementary Information**

**Canada’s Medical Assistance in Dying system can enable healthcare serial killing**

*HEC Forum*

**Table 3 Indicators and enablers of serial murder in healthcare settings** (adapted from Crofts, 2022; Frank, 2020; Lubaszka et al., 2014; Tang, 2020; Tilley et al., 2019; Yardley & Wilson, 2016; Yorker et al., 2006)

| **Characteristics of healthcare serial killing and killers** | **Presence or analogue in MAiD** |
| --- | --- |
| **Types**  (Miller, 2014) | |
| 1. Custodial | All euthanasia-MAiD providers commit custodial homicides, distinguished as culpable and non-culpable status relative to their compliance with criminal law. |
| 1. Delusional/Mission | MAiD is framed as mission on a spectrum of enforcing an ideological positive constitutional or human right to die, or a clinical procedure to end or relieve extreme suffering through death.  Some providers flatly deny they kill people or what they do euthanasia or assisted suicide.  Some providers describe MAID as a crusade, empowerment, and social justice. |
| 1. Utilitarian | The billable nature is a financial incentive to maximise assessments and provisions and MAiD is the main practice and thus a major income source for some providers.  Ending of suffering with death is a key MAiD rationale. Personal eugenical beliefs among clinicians are unknown. |
| 1. Sexual/sadistic | Reactions from MAiD providers include adrenaline highs and feeling good, sexual arousal, and laughing while recounting distress in witnesses and patients. |
| 1. Mercy-hero (Soria & Ansa, 2016) | Where MAiD is described by compassionate relief of suffering without references to euphoria or arousal associated with sadism. |
| **Indicators** | |
| 1. IV or injection to administered lethal doses of routine medical substances | MAiD by euthanasia is the IV or syringe injection of lethal overdoses of common medical substances. While self-administration is legally permitted outside Quebec, some Canadian provinces and jurisdictions only offer euthanasia, accounting for 99.95% of MAiD deaths. |
| 1. Employment a history involves unusual number of job changes, complaints, sanctions, restrictions, etc. | Clinicians are not vetted.  High level complaints exist against MAiD clinicians regarding ineligible or problematic deaths. While none are known to have resulted in discipline, a history of complaints is unusual. |
| 1. Personal history may involve substance abuse or interpersonal difficulties, mental illness, or disorders, especially personality related | Clinicians are not vetted.  At least one provider admits childhood and family interpersonal problems. |
| 1. Attention craving, arrogant, or enthusiastic about skills | Some MAiD clinicians  - express self-promotional accounts in books and media and make strong claims about the arguable sufficiency of existing safeguards  - express confidence in their informal ability to assess patient capacity or judge suicidality without drawing on expertise or recognised tests  - provide MAiD in controversial settings like religious facilities against requests - define MAiD on personal terms instead of accepted clinical or legal concepts and rationales. |
| 1. Use of neutralisation techniques to justify or disown responsibility for killings and resulting harm | Providers frame MAiD as “care” not euthanasia or suicide, blame the state for failing to provide enough support to patients necessitating MAiD, helping people in “great need” of death, and as peaceful or dignified death, or blame the (potentially non-terminal) disease or illness for killing the patient, not their lethal injection.  Trauma to witnesses may be minimised or deflected as unrelated to MAiD. |
| 1. Suspicious or alarmed colleagues | Some providers and other clinicians have publicly stated concerns. Provincial Chief Medical Officers, Coroners, and a provincial overseer have made complaints and warnings. Internal complaints and warnings are will not be publicly known. |
| 1. Actions claimed as merciful, not malicious | Claims of merciful relief of suffering are a key rationale for MAiD. MAiD providers state they are providing ‘care’ and Québec MAiD commission warned about claiming ‘care’ not euthanasia as reason for increased rates. |
| 1. Nicknames like ‘angel of death’ | Unknown and requires access to complaint records or interviews with colleagues. |
| 1. Predictions of patient deaths or prognosis | Assumption of prognostic power are inherent the eligibility criteria of irremediable and foreseeable - especially uncertain in mental illness.  CAMAP material implies that clinicians can prognose death from patients’ “intention” to attempt suicidal refusals of care. |
| 1. Inconsistencies in accounts of deaths | Accounts by clinicians and families may differ.  Some providers also find rejected patients eligible after assessor shopping. |
| 1. Secrets medicines at work and home | Legitimate access to medicines for MAiD means that providers do not need to illicitly possess them.  A veterinarian reported working as a MAiD provider may have access to euthanasia drugs that that avoid evidence the trail in pharmacies and hospitals.  At least one region permits family members to administer the lethal injections. |
| 1. Higher instances of death on a shift | Wide variation in the to-date distribution of deaths per provider. Some providers have killed hundreds of patients, others just one.  A small number of providers appear to be responsible for most MAiD deaths (<336/1837 in 2022). |
| 1. Victims are ill, elderly, or disabled | Patients eligible for MAiD match common victim profiles of HSK, e.g., elderly, women, or physically ill (or with comorbid mental illness). |
| 1. Predation toward patients unable to resist or lacking mental or physical capacity | MAiD capacity assessments are rare and some clinicians appear to avoid assessments or seek ways to reduce capacity.  Reports imply some providers may actively try to recruit MAiD patients. |
| 1. Opportunity seeking where they won’t be easily observed | MAiD assessments tend to occur in private and what is said is only recorded by the clinician. |
| 1. Poor clinic and system level surveillance to detect anomalies in the number of types of deaths or other adverse medical events | Poor surveillance of MAiD deaths and practitioners due to lack of national standards, local variation in health administration, coroner reporting requirements, and data collection. Poorly presented report data from Health Canada reports and limited data collection can conceal anomalies. |
| 1. Reports or complaints from patients, colleagues, family members, or other observers about problems | Quebec identified possibly culpable homicides by providers and warned about lawbreaking.  Numerous complaints have filed against providers by family members, clinical managers, and senior oversight officials but so far none is known to have succeeded. |
| 1. Poor record keeping | Reporting requirements for MAiD deaths vary by province and territory in terms of documentation and recordkeeping, without a meta-regulator to collate data.  How MAiD is recorded on Medical Certificates of Death and the level, if any, of coroner involvement is also inconsistent.  Oversight is inconsistent and assessors and providers themselves are responsible for the contents of much documentation. |
| **MAiD specific issues** | |
| 1. Data deficient and ambiguous official reporting | Federal reports of MAiD cases contain omissions and poor quality data that could conceal non-compliance with the law.  Non-compliance, assessor shopping, and other data are not tracked. |
| 1. Variations in reporting deaths | Reporting requirements for MAiD coroners vary by province and territory.  Some most MAiD deaths are not reported to coroners (e.g., British Columbia).  Non-compliance with law is self-reported.  MAiD not always recorded death certifications. |
| 1. Broad criminal exemptions for MAiD clinicians | MAiD provides exemptions to criminal culpability for assessors, providers, and other clinical staff, making identification and prosecution of criminal homicide more challenging compared to non-MAiD healthcare serial murder. Historic and present day admissions of illegal provision or documented offences (non-compliant deaths, family administration) remain unprosecuted. |
| 1. Lack of uniform assessment and provision standards | Assessments lack uniform standards and are unsupervised. Some assessors are state they use personally concocted non-clinical ‘litmus tests’ outside of Criminal Code eligibility criteria to approve ‘borderline’ patients. |
| 1. Ideology | MAiD rationales vary from constitutional right to extreme end-of-life treatment, which may influence assessments. |
| 1. Post-mortem patient privacy law | Privacy law may be used by local health authorities to block family and law enforcement scrutiny of medical records when they raise questions about MAiD deaths. |
| 1. Assessor shopping | Patients deemed ineligible by other providers may seek unlimited further assessments increasing the likelihood they will intersect with a clinician more willing to find ways to approve and provide for them. |

Crofts, P. (2022). Gosport Hospital, euthanasia and serial killing. In D. J. Fleming & D. J. Carter (Eds.), *Voluntary assisted dying: Law? Health? Justice?* (pp. 155–178). Australian National University Press.

Frank, C. (2020). Health care serial murder: What can we learn from the Wettlaufer story? *Canadian Family Physician*, *66*(10), 719–722.

Lubaszka, C. K., Shon, P. C., & Hinch, R. (2014). Healthcare Serial Killers as Confidence Men. *Journal of Investigative Psychology and Offender Profiling*, *11*(1), 1–28. https://doi.org/10.1002/jip.1394

Miller, L. (2014). Serial killers: I. Subtypes, patterns, and motives. *Aggression and Violent Behavior*, *19*(1), 1–11. https://doi.org/10.1016/j.avb.2013.11.002

Soria, M. Á., & Ansa, N. (2016). Psychological Motivational Profile of a Serial Killer “Mercy-Hero” vs. Power/Control Type. *Journal of Psychology and Psychotherapy Research*, *3*(1). https://doi.org/10.12974/2313-1047.2016.03.01.2

Tang, F. (2020). *A qualitative exploration into the subjective experiences of healthcare serial killers.* [Master Thesis, Wilfred Laurier University]. https://scholars.wlu.ca/etd/2279

Tilley, E., Devion, C., Coghlan, A. L., & McCarthy, K. (2019). A Regulatory Response to Healthcare Serial Killing. *Journal of Nursing Regulation*, *10*(1), 4–14. https://doi.org/10.1016/S2155-8256(19)30077-8

Yardley, E., & Wilson, D. (2016). In Search of the ‘Angels of Death’: Conceptualising the Contemporary Nurse Healthcare Serial Killer. *Journal of Investigative Psychology and Offender Profiling*, *13*(1), 39–55. https://doi.org/10.1002/jip.1434

Yorker, B. C., Kizer, K. W., Lampe, P., Forrest, A. R. W., Lannan, J. M., & Russell, D. A. (2006). Serial Murder by Healthcare Professionals. *Journal of Forensic Sciences*, *51*(6), 1362–1371. https://doi.org/10.1111/j.1556-4029.2006.00273.x
